# Supplementary material for: Aberrant Auditory and Visual Memory Development of Children with Upper Limb Motor Disorders
Source: Brain Sci. 2021 Dec 15;11(12):1650. doi: 10.3390/brainsci11121650 (PMC8699193; doi:10.3390/brainsci11121650)
Supplement: Supplementary file 1 [file brainsci-11-01650-s001.zip › brainsci-1491907-supplementary.pdf]

Supplementary materials.

	AVERAGE AGE			
**AGE**	**Children with motor disorder**		**Control group**	
3 - 15 y.o.	8,3		9,6	
	**MALE**	**FEMALE**	**MALE**	**FEMALE**
3 - 15 y.o.	8,2	8,4	9,2	10,1
	**Children with motor disorder**		**Control group**	
3 - 6 y.o.	4,7		5,2	
7 - 10 y.o.	8,8		8,5	
11 - 15 y.o.	13,0		12,5	

Supplementary Table S1. Average values by age of children with motor disorders and the control group.

Group	Age period	Children with motor disorder	Control group
**Group A**	3–6 years old	22 children	6 children
**Group B**	7–10 years old	24 children	13 children
**Group C**	11–15 years old	11 children	13 children

Supplementary Table S2. Visual representation of the division into groups by age of children with motor disorders and the control group.

	Patients 3-7 years		8-10 years		11-15 years		Controls 3-7 years		8-10 years		11-15 years	
	Mean	SD	Mean	SD	Mean	SD	Mean	SD	Mean	SD	Mean	SD
Attention Span	2.27	0.91	3.32	1.30	4.00	1.34	2.25	1.09	4.20	0.75	4.43	1.05
Auditory Memory	3.55	1.16	4.74	1.45	6.00	1.55	4.75	1.09	6.20	0.75	6.29	0.88
Visual Memory	4.14	1.42	5.00	1.38	6.80	1.89	5.75	1.98	7.00	0.63	7.29	1.03
Intelligence	2.32	0.87	2.68	0.92	2.80	1.08	2.50	0.87	2.20	0.40	2.43	1.40
Storytelling	2.36	1.15	3.47	1.09	3.10	1.04	3.12	0.60	3.00	0.63	3.57	0.49
Thinking	4.77	1.65	5.32	1.08	5.70	1.19	6.50	0.71	7.00	0.00	6.29	1.16
ACS	-0.57	0.49	0.05	0.48	0.43	0.49	0.04	0.28	0.52	0.20	0.63	0.21

Supplementary Table S3. Means and standard deviation (SD) of scores for both groups of children in all age ranges

	Age F (1, 64)	p	η_p^2	q	Group F (1, 64)	p	η_p^2	q	Sex F (1, 64)	p	η_p^2	q
Attention Span	38.80	< 0.001 ***	0.37	< 0.001	2.89	0.09	0.04	0.13	1.15	0.29	0.02	0.50
Auditory Memory	37.92	< 0.001 ***	0.36	< 0.001	12.74	< 0.001 ***	0.16	< 0.01	0.46	0.50	0.01	0.58
Visual Memory	23.57	< 0.001 ***	0.26	< 0.001	16.19	< 0.001 ***	0.19	< 0.001	2.11	0.15	0.03	1.00
Intelligence	0.81	0.37	0.01	0.37	0.33	0.57	0.00	0.57	1.80	0.18	0.03	0.43
Storytelling	8.97	< 0.01 **	0.12	< 0.01	1.45	0.23	0.02	0.27	1.94	0.17	0.03	0.59
Thinking	5.24	< 0.05 *	0.07	< 0.05	17.28	< 0.001 ***	0.21	< 0.001	0.71	0.40	0.01	0.56
ACS	58.43	< 0.001 ***	0.47	< 0.001	20.84	< 0.001 ***	0.24	< 0.001	0.03	0.87	0.00	0.87

Supplementary Table S4. Statistical results for cognitive performance (ANCOVA) as a function of factors Group (df = 1; either patient or control), Sex (df = 1; either male or female) and Age (continuous).
